# Infantile hepatic hemangiomas associated with high-output cardiac failure and pulmonary hypertension

**DOI:** 10.1186/s12872-019-1200-6

**Published:** 2019-10-11

**Authors:** Xin-tong Zhang, Wei-dong Ren, Guang Song, Yang-jie Xiao, Fei-fei Sun, Nan Wang

**Affiliations:** Department of Ultrasound, Shengjing Hospital of China Medical University, sanhao street 36#, Shenyang, 110004 CN China

**Keywords:** Infantile hepatic hemangioma, Pulmonary hypertension, Congestive heart failure, Congenital heart disease

## Abstract

**Background:**

Infantile hepatic hemangioma (IHH) is a rare endothelial cell neoplasm, which may be concurrent with severe complications and result in poor outcomes. Moreover, the coexistence of IHH and congenial heart disease is even rarer.

**Case presentation:**

We present a 10-day-old male born with IHH associated with patent ductus arteriosus (PDA), atrial septal defect (ASD) and pulmonary hypertension. Moreover, we reviewed a series of studies of IHH-associated high-output cardiac failure between 1974 and 2018, and summarized the treatment outcomes.

**Conclusions:**

Infantile hepatic hemangioma (IHH) has been known to induce high-output heart failure. There is no literature to summarize the severity of its impact on heart, which can lead to a high mortality rate. When IHH is detected by ultrasound, the heart should be evaluated to facilitate treatment. The outcomes of IHH associated with heart failure are good.

## Background

Infantile hepatic hemangioma (IHH) is a rare proliferative endothelial cell tumor. It appears to be a benign tumor, however, it may lead to poor outcomes because of severe complications such as congestive heart failure (CHF), which occurs in 15% of infants with this disease [1]. More rarely, IHH is concurrent with congenital heart disease (CHD). Herein we present a patient with IHH, patent ductus arteriosus (PDA), and atrial septal defect (ASD). Meanwhile, we review and summarize the injury of IHH on the heart, and related outcomes.

## Case presentation

A 10-day-old boy was born at 38 weeks’ gestation and had tachypnea at 65 breaths per minute. The liver margin was palpable 4 cm below the left costal margin. The heart rhythm was normal but a grade II-IV systolic murmur could be heard. Laboratory tests showed that his brain natriuretic peptide (BNP) level was greater than 5000 pg/ml. His CKMB was 110 U/L, C-reactive protein was 23.4 mg/L, alpha fetoprotein was greater than 1210 ng/ml, PTA 54%, INR 1.6, APTT 50 s, FIB 1.5 g/l, D-dimer 1064 μg/L, HGB, 123 g/L, MCH 36.4 pg, total bilirubin (BilT)196.7 umol/L, BilD 13.2 umol/L, ALT 44 U/L, and AST 23 U/L. Blood gas analysis revealed that the carbon dioxide pressure was raised to 53.4 mmHg and the oxygen partial pressure was 62.5 mmHg.

**Fig. 1:**
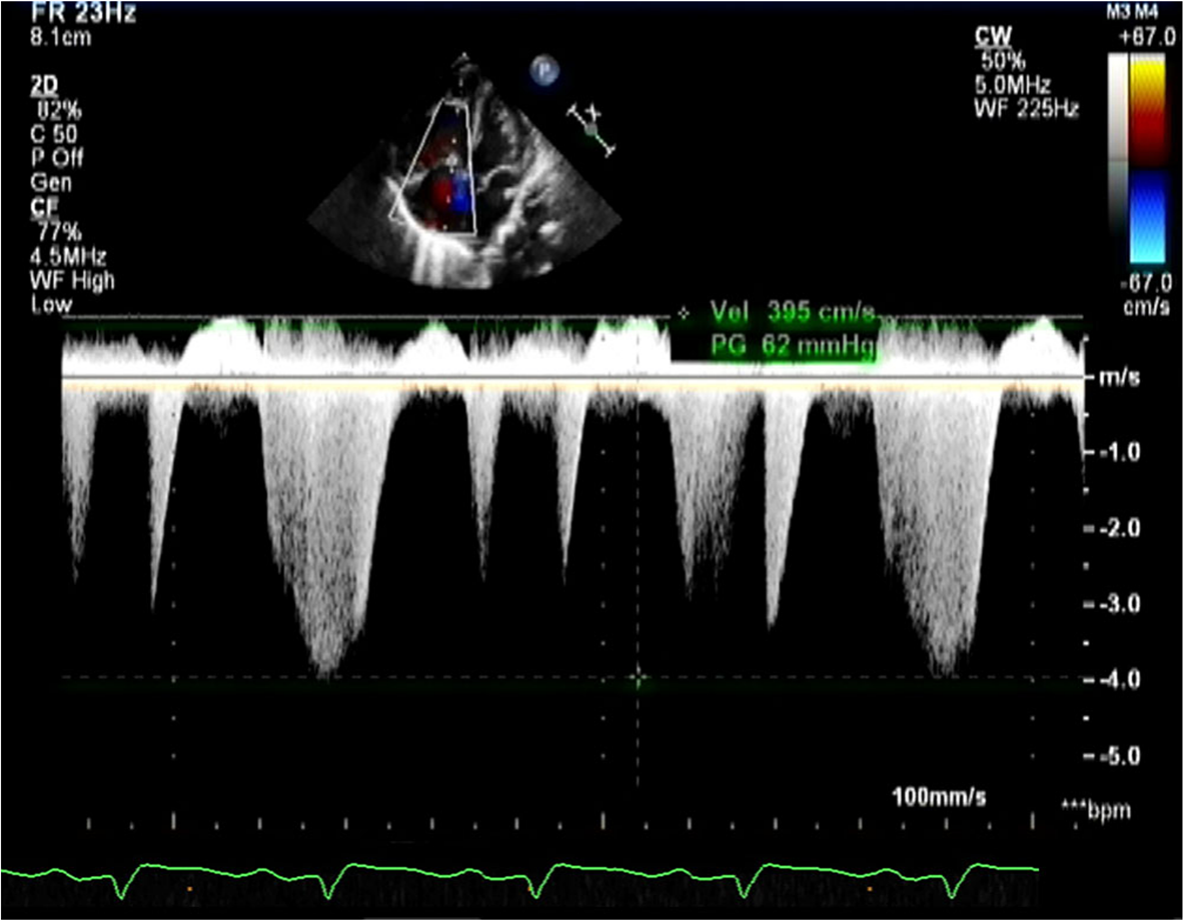
IHH (Infantile hepatic hemangiomas) on abdominal ultrasound, in the left hepatic lobe. CDFI(Color doppler flow imaging) can be detected with abundant blood flow signals

**Fig. 2:**
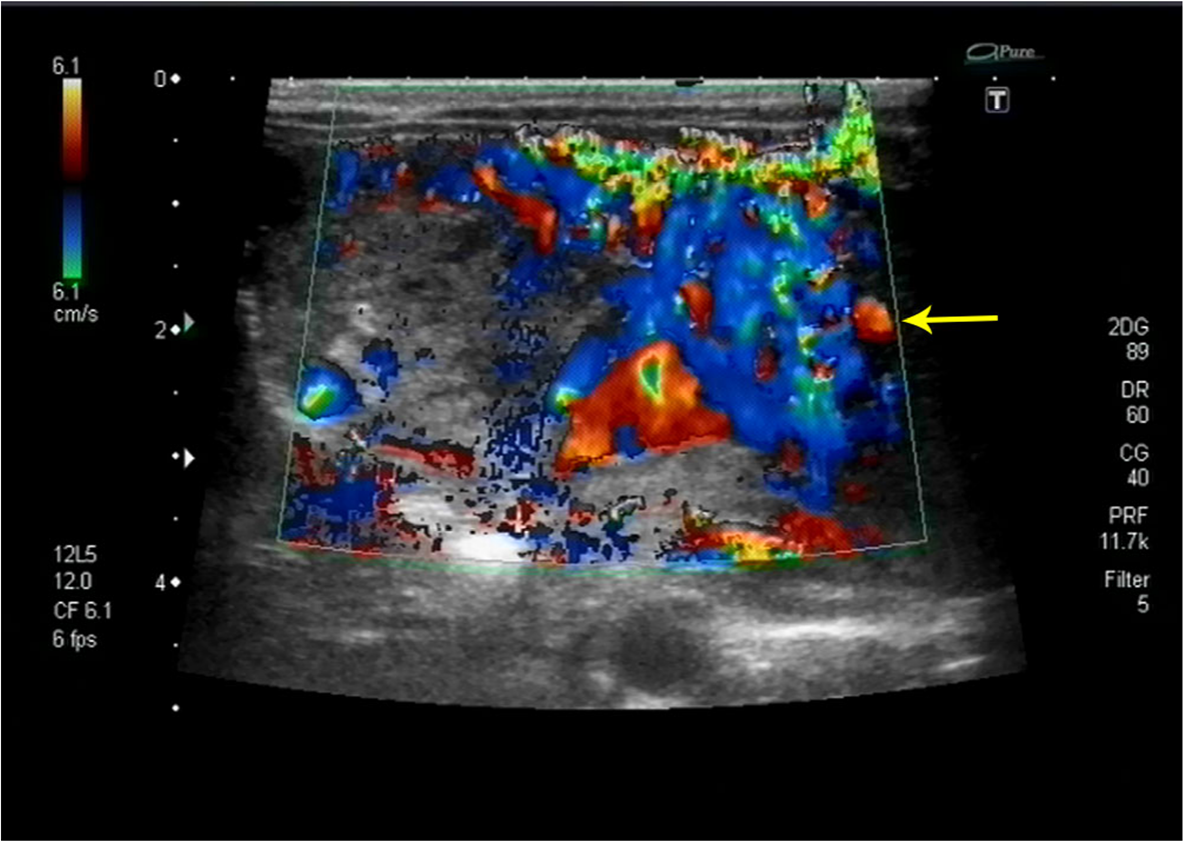
Views of the IHH from contrast-enhanced CT (Contrast-enhanced computed tomography). The left hepatic lobe is irregularly enhanced with a slightly lower density and the lesion shows peripheral enhancement of centripetal fill-in on arterial phase

**Fig. 3:**
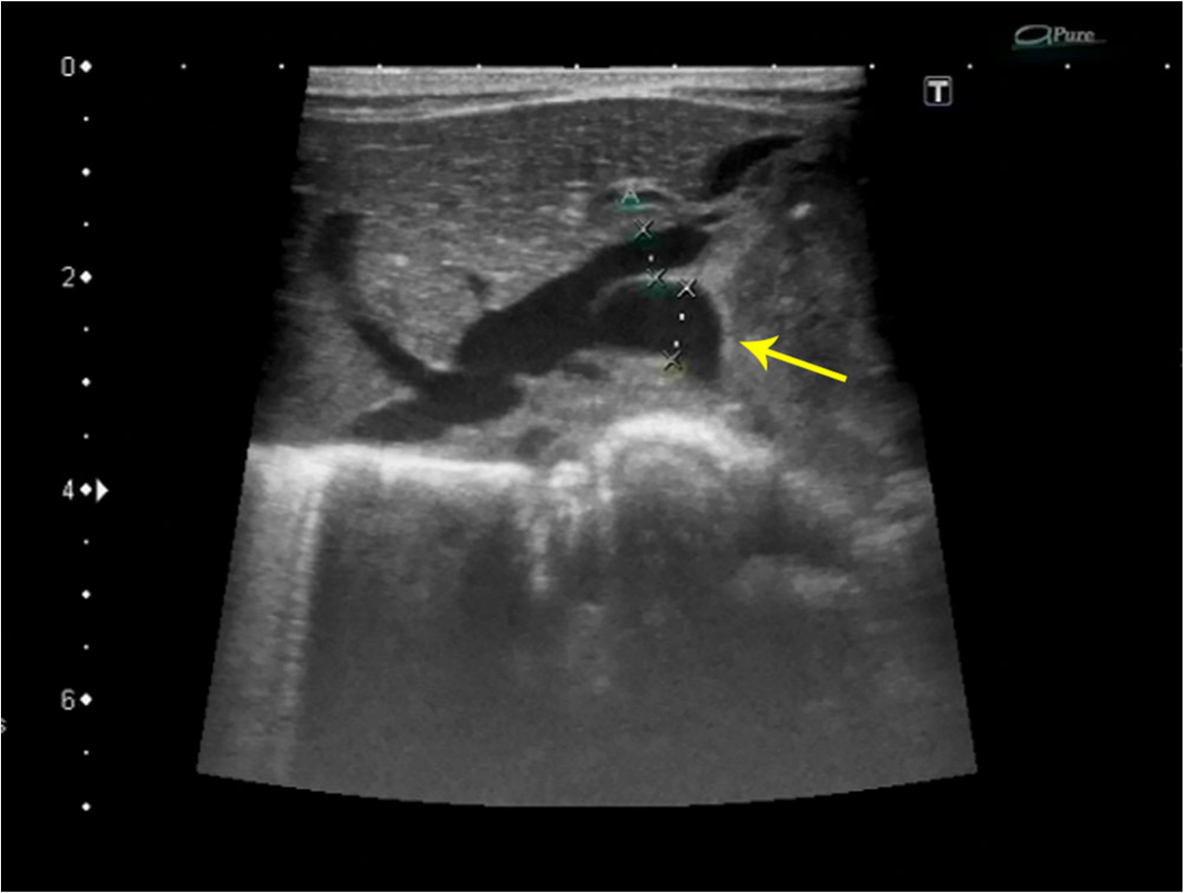
Expansion of the left hepatic vein close to the lesion

Echocardiography demonstrated a small PDA of 1.5-1.8 mm with right to left shunting, a large ASD with left to right shunting, and severe right atrial and right ventricular enlargement. A severe systolic pressure gradient of 70 mmHg suggested marked elevation of pulmonary artery pressure at the near systemic level. (Fig. 1) Color Doppler scanning of the liver displayed abundant blood flow in the lesion. (Fig. 2) The left hepatic vein was dilated to 8 mm with two great branches feeding the mass. (Fig. 3)The right hepatic artery and a branch arising from the abdominal aorta were also in close association with the lesion.

**Fig. 4:**
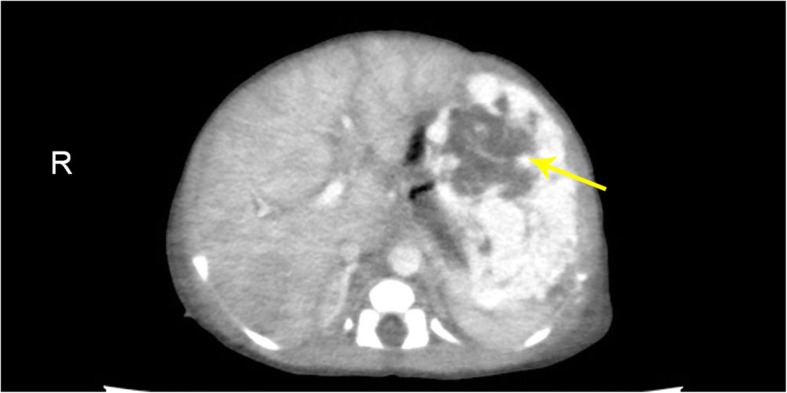
View of the patient’s moderate to severe pulmonary arterial pressure

Contrast-enhanced computed tomography (CT) showed that the lesion enhanced irregularly in the left lobe of the liver. (Fig. 4)It was irregularly hypodense on plain scan with peripheral enhancement in the arterial phase and centripetal fill-in in the portal venous phase. In the delayed phases, the density of the leision was slightly higher than that of the liver parenchyma**.** The patient received diuretic therapy, fluid restriction, low-flow oxygen, and infection control for the management of the heart failure. After treatment his breathing difficulty improved. Then the patient was transferred to Beijing Children’s Hospital for surgery and the adhesion between the tumor and the intestine was found during the operation. The patient eventually died due to postoperative complications and multiple organ failure.

## Discussion and conclusions

IHH is an endothelial cell neoplasm, a benign tumor, which is usually clinically silent and slowly progressive during childhood. Although almost all are asymptomatic, a small subset can produce high-output cardiac failure and cause considerable mortality. Its pathologic changes are similar to that of hepatic artery to hepatic vein or hepatic artery to portal vein arteriovenous fistula. The prognosis of the disease is poor when complications present and the mortality rate can be as high as 90% [2, 3]. We reviewed a series of recent studies of IHH-associated high-output cardiac failure between 1974 and 2018, and summarized the treatment outcomes.

Heart failure was obviously a clinical relevant complication in the 25 cases which were presented in Table 1 [2, 4–22]. The age of diagnosis varied ranging from 1 day to 3.5 years. Eight patients (28%) presented with pulmonary hypertension including two mild, one moderate, three severe, and two unknown. Two of them were also associated with other congenital cardiac malformations which were illustrated in detail in Table 2 [7, 11, 13, 22–24]. Cardiac function improved after treatment in 15 patients, achieving normal value in 2. Consequently the outcomes of IHH-associated high-output cardiac failure proved to be quite satisfying. Most of the patients discharged or achieved remarkable improvement through appropriate treatment, while only 4 of them failed to survive.

**Table 1:** Summary of the literature on patients presenting with IHH associated with congestive heart failure

Study	Age	Sex	Cardiopathy	Diagnose methods	Treatment	Cardiac recovery	Outcome
Mattioli et al. (1974) [16]	27d	F	BVH, PAH	HAG	Ligation	Normal	Discharged
Linderkamp et al. (1976) [14]	1d	M	CHF	Renal scan	Resection	Improve	Discharged
Rotman et al. (1980) [18]	4 m	F	CHF	VG	Glucocorticoid	Improve	Remarkable improvement
Burke et al. (1986) [2]	3.5y	F	Cardiomegaly	US,CT	Embolization	–	Failure
Gozal et al. (1990) [9]	17d	F	Cardiomegaly	US	Glucocorticoid	Improve	Discharged
Kristidis et al. (1991) [13]	3d	M	ASD,BVH,PDA	US	Prednisone	Improve	Discharged
	3d	M	CHF	US	Prednisone,digoxin	Improve	Discharged
	1d	F	CHF	US	Prednisone	Improve	Discharged
Barsever et al. (1994) [4]	2w	F	CHF cardiomegaly	US,CT	Interferon	–	Remarkable improvement
Hazebroek et al. (1995) [10]	2d	M	CHF	US,HAG	Ligation	Improve	Remarkable improvement
Fok et al. (1996) [8]	1d	M	CR = 0.77, CHF	US,TRA	Embolization	–	Discharged
Lu C C et al. (2002) [15]	1d	F	CR = 0.8	US,CT	Glucocorticoid,dopamine	------------	Remarkable improvement
	10d	M	BVH	US,MRI	Ligation	Improve	Discharged
Sakamoto et al. (2010) [19]	4d	F	CHF	CT	Transplantation	–	Remarkable improvement
Mhanna et al. (2011) [17]	8w	F	CHF	US,CT	Glucocorticoid,propranolol	------------	Discharged
	3 m	M	ASD,CHF,LVH,PDA	US	Glucocorticoid,propranolol	------------	Discharged
Dotan et al. (2013) [7]	11w	F	BVH,PAH	US	propranolol	Improve	Discharged
Dasgupta et al. (2013) [6]	1d	M	BVH	US,CT	Glucocorticoid	Improve	Remarkable improvement
Chopra et al. (2014) [5]	9 m	F	CHF	US,CT	Glucocorticoid	–	Failure
Ye et al. (2014) [14]	59d	F	PAH,RVH	US,MRI	Surgery	–	Failure
Imai et al. (2015) [12]	1d	M	Cardiomegaly	MRI,CT	Glucocorticoid	Improve	Discharged
Wang et al. (2015) [24]	5 m	F	Cardiomegaly,PA	US,CT	Embolization	Improve	Discharged Failure
	19d	F	H BVH,PAH	US,CT	Embolization	Improve	
Shen et al. (2016) [20]	11d	M	CHF	US	Glucocorticoid, Embolization	Normal	Discharged
Hutchins et al. (2017) [11]	22 m	F	VSD	US,CT,MRI	Glucocorticoid,Sirolimus	–	Discharged

*BVH* Biventricular hypertrophy, *RVH* Right ventricular hypertrophy, *“——“* No information available, *CR* Cardiothoracic ratio, *VG* Venacavography, *HAG* Hepatic arterograph

**Table 2:** Summary of patients presenting with IHH associated with pulmonary artery hypertension

Study	Sex	Age	EF(%)	Heart	TR	PAH mmHG	PDA	VSD,ASD
Present study	M	10d	72	RHE	moderate	70	1.5-1.8 mm R-L	ASD 7.5 mm L-R
Wang et al. (2015) [24]	F	5 m	78	cardiomegaly	–	54	–	–
Kristidis et al. (1991) [13]	F	19d 3d	64	RHE cardiomegaly	severe	90 PAH	--	--
	F		--		--		--	Small ASD L-R
Dotan et al. (2013) [7]	F	11w	normal	BVH	mild to moderate	58	–	–
Ye et al. (2014) [22]	F	59d	–	RHE	–	120	–	–
Ersch et al. (2002) [23]	F	20 m	–	RHE	–	PAH	–	–
Hutchins et al. (2017) [11]	F	22 m	–	RHE	mild to moderate	70	–	Small VSD

*RHE* Right heart enlargement, *BVH* Biventricular hypertrophy, *PDA* Patent ductus arteriosus, *R-L* Right to left shunt, *L-R* Left to right shunt, *TR* Tricuspid regurgitation, −- No information available

IHH can be classified as focal, multifocal, or diffuse [9]. The diagnosis of IHH relies on ultrasonography (US), CT, and magnetic resonance imaging (MRI). CT and MRI can reveal discrete lesions in patients. Diffuse lesions require baseline determination of size, cardiac and thyroid function, and coagulation profile. When IHH is associated with heart disease, cardiac structure and function can be observed by echocardiography, which can identify intra- or extracardiac disease [3]. There were 13 (52%) patients in our series that were diagnosed by echocardiography. In our case, the patient had a focal lesion, which is diagnosed primarily by ultrasonography and CT.

The pathological mechanism of CHF in IHH is associated with arteriovenous shunts in hemangiomas. The arteriovenous shunts result in a decrease of systemic blood volume as well as increase of pulmonary blood volume, thus leading to the cardiac output increase. Furthermore, aggravated by the pulmonary hypertention it finally leads to high-output CHF [21]. In fetal stage, high pulmonary vascular resistance and pulmonary pressure help maintain the fetal circulation, however, after birth the high pulmonary pressure will descend gradually within 3 months while systemic pressure of neonates will ascend with closure of oral foramen. Nevertheless, the existence of IHH can increase the load of right heart system and affect circulation transition from fetus to neonate, which further increase pulmonary vascular resistance and cause pulmonary hypertention.

Various therapies have been reported to treat IHH, including drugs, embolization, ligation, and resection [25]. (1) Drugs: steroid therapy functioned well in improving hemodynamics, reducing hepatic vascularity as well as deferring early emergency delivery in congestive heart failure fetus as recorded in literatures. There are no significant differences between single or combined drug use in the literature. (2) Embolization: embolization has been strongly suggested for provisional stabilization of fatal congestive heart failure combined with pharmacological therapy. (3) Ligation: ligation can reduce oxygen supply to hepatocytes and improve liver function. (4) Resection: surgery should be considered when medical management failed [3].Treatment methods and their outcomes as described in the literature are shown in Table 3. Among 25 patients, four ended in treatment failure (18%).

**Table 3:** Demographics for treatment outcomes

IHH	All	Success	Failure
Embolization	5	3	2
Ligation	3	3	0
Resection	1	1	0
Transplantation	1	1	1
Drugs:	10	9	1
Glucocorticoid propranolol	2	2	0
Glucocorticoid dopamine	1	1	0
Prednisone digoxin	1	1	0
Glucocorticoid	5	4	1
Propranolol	1	1	0
Prednisone	2	2	0
Interferon	1	1	0
Glucocorticoid,Sirolimus	1	1	0
	25	21	4

Success = remarkable improvement or discharged

Early age of onset is typical of IHH with heart failure. When IHH is detected by US, echocardiography should also be performed timely for more detailed information about cardiac structure and function. For infants in life-threatening and complicated conditions, US and echocardiography should be performed as early as possible to evaluate IHH associated with congestive heart failure and to facilitate treatment therapies. With regard to the treatment in our review, outcomes of IHH with heart failure are considered to be good.

## Data Availability

All data that was generated or analyzed during the current study are available from the corresponding author on reasonable request.

## Abbreviations

ALT: Alanine aminotransferase
APTT: Activated partial thromboplastin time
ASD: Atrial septal defect
AST: Aspartate aminotransferase
BilD: Direct bilirubin
BilT: Total bilirubin
BNP: Brain natriuretic peptide
CHF: Congestive heart failure
CKMB: MB isoenzyme of creatine kinase
PTA: Prothrombin time activity
CT: Computed tomography
FIB: Fibrinogen
HGB: Hemoglobin
IHH: Infantile hepatic hemangioma
INR: International normalized ratio
MCH: Mean corpuscular hemoglobin
MRI: Magnetic resonance imaging
PDA: Patent ductus arteriosus
US: Ultrasonography
